# Supplementary material for: Structural basis for GSDMB pore formation and its targeting by IpaH7.8
Source: Nature. 2023 Mar 29;616(7957):590–7. doi: 10.1038/s41586-023-05832-z (PMC10115629; doi:10.1038/s41586-023-05832-z)
Supplement: Supplementary file 1 — This file contains the uncropped immunoblots (Supplementary Figs. 1 and 2). [file 41586_2023_5832_MOESM1_ESM.pdf]

nature portfolio

https://doi.org/10.1038/s41586-023-05832-z

Supplementary information

Structural basis for GSDMB pore formation and its targeting by IpaH7.8

In the format provided by the authors and unedited

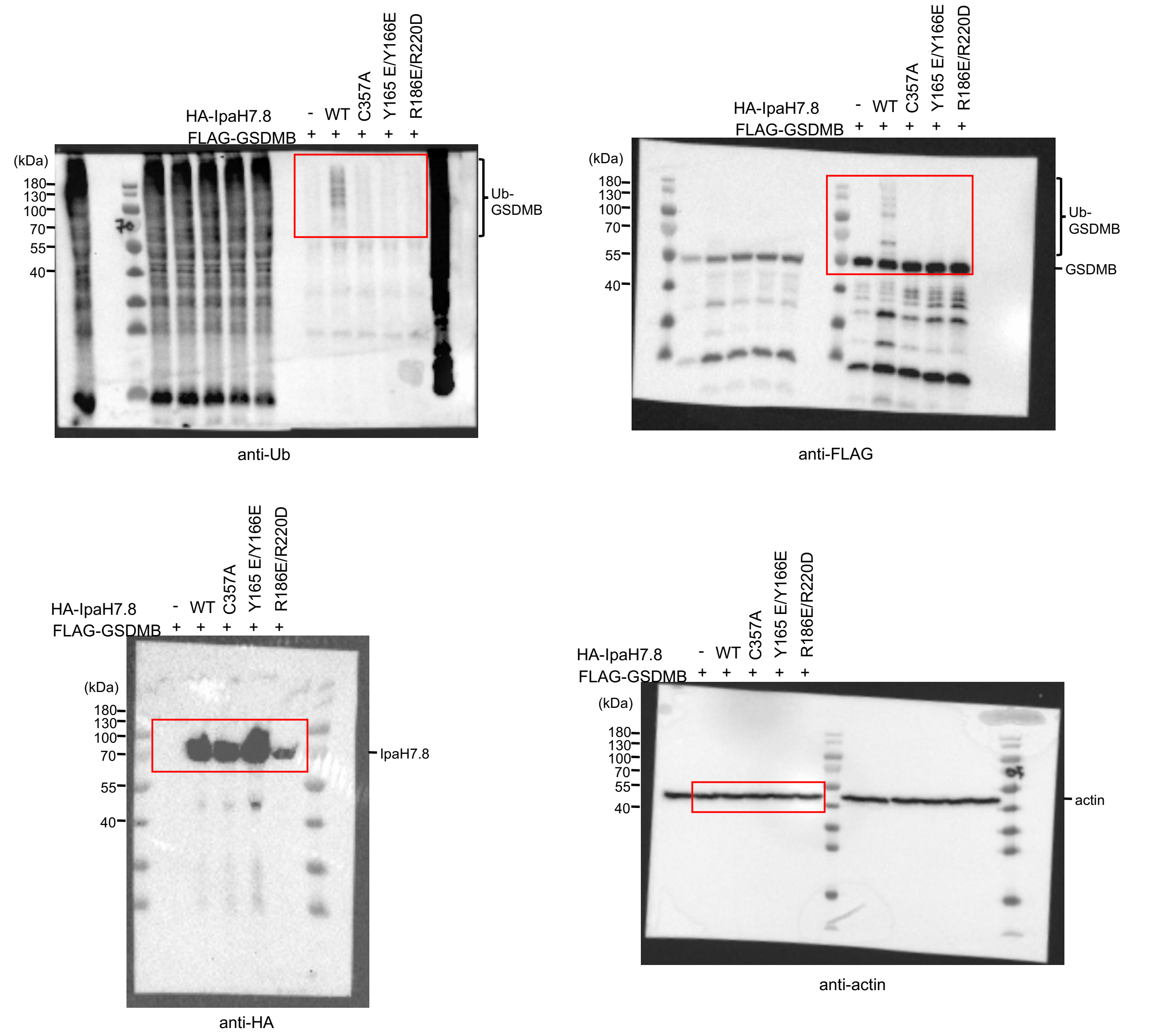

SI Figure 1 | Uncropped immunoblots from Figure 2f. Immunoblots for Fig. 2f showing the ubiquitination of GSDMB in HEK293T cells co-transfected with FLAG-tagged GSDMB and HA-IpaH7.8 (WT or indicated mutants). Proteins were detected by western blotting, using the indicated antibodies and the red rectangles show the cropping location.

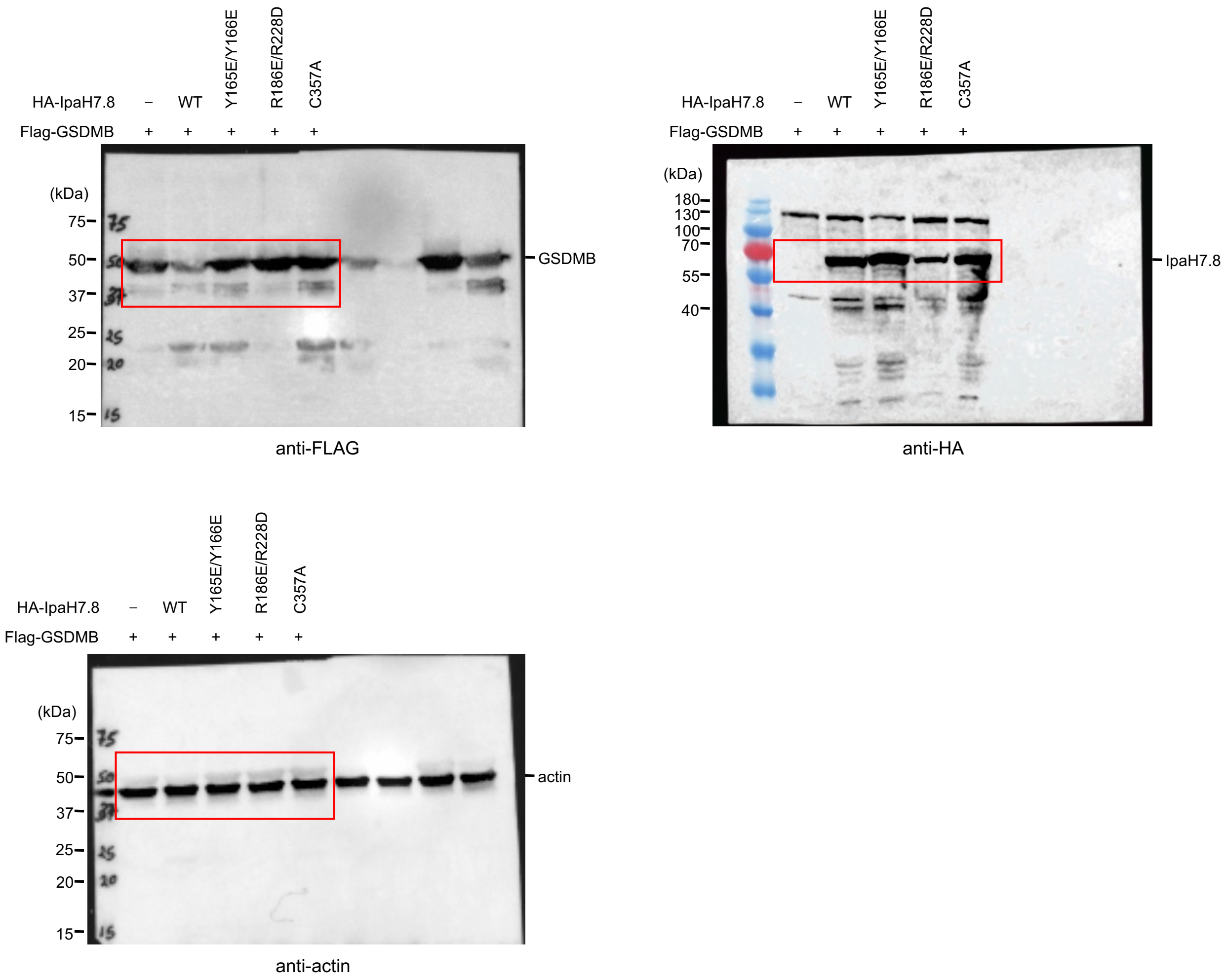

SI Figure 2 | Uncropped immunoblots from Extended Data Fig. 3c. Immunoblots for Extended Data Fig. 3c showing the protein level of FLAG-tagged GSDMB in 293T cells when co-transfected with HA-IpaH7.8 (WT or indicated mutants). Proteins were detected by western blotting, using the indicated antibodies and the red rectangles show the cropping location.
