# Supplementary material for: Structural basis for GSDMB pore formation and its targeting by IpaH7.8
Source: Nature. 2023 Mar 29;616(7957):590–7. doi: 10.1038/s41586-023-05832-z (PMC10115629; doi:10.1038/s41586-023-05832-z)
Supplement: Supplementary file 2 — Reporting Summary [file 41586_2023_5832_MOESM2_ESM.pdf]

nature portfolio

Corresponding author(s): Jianbin Ruan

Last updated by author(s): Jan 27, 2023

Reporting Summary

Nature Portfolio wishes to improve the reproducibility of the work that we publish. This form provides structure for consistency and transparency in reporting. For further information on Nature Portfolio policies, see our Editorial Policies and the Editorial Policy Checklist.

Statistics

For all statistical analyses, confirm that the following items are present in the figure legend, table legend, main text, or Methods section.

n/a	Confirmed	
☐	☒	The exact sample size (*n*) for each experimental group/condition, given as a discrete number and unit of measurement
☐	☒	A statement on whether measurements were taken from distinct samples or whether the same sample was measured repeatedly
☐	☒	The statistical test(s) used AND whether they are one- or two-sided<br>*Only common tests should be described solely by name; describe more complex techniques in the Methods section.*
☒	☐	A description of all covariates tested
☒	☐	A description of any assumptions or corrections, such as tests of normality and adjustment for multiple comparisons
☐	☒	A full description of the statistical parameters including central tendency (e.g. means) or other basic estimates (e.g. regression coefficient) AND variation (e.g. standard deviation) or associated estimates of uncertainty (e.g. confidence intervals)
☒	☐	For null hypothesis testing, the test statistic (e.g. *F*, *t*, *r*) with confidence intervals, effect sizes, degrees of freedom and *P* value noted<br>*Give P values as exact values whenever suitable.*
☒	☐	For Bayesian analysis, information on the choice of priors and Markov chain Monte Carlo settings
☒	☐	For hierarchical and complex designs, identification of the appropriate level for tests and full reporting of outcomes
☒	☐	Estimates of effect sizes (e.g. Cohen's *d*, Pearson's *r*), indicating how they were calculated

Our web collection on statistics for biologists contains articles on many of the points above.

Software and code

Policy information about availability of computer code

Data collection	We used SerialEM for cryo-EM data collection.
Data analysis	We used MotionCor2, CTFFIND4, Relion 4.0, cryoSPARC v3.3.1, crYOLO 1.7.6, Coot 0.9.8.4, PHENIX 1.20-4487, PyMol 2.5.3, ResMap 1.95, Molprobity 4.02-528 and UCSF Chimera 1.15 for cryo-EM data analysis, and used Origin 7.0 for ITC data analysis

For manuscripts utilizing custom algorithms or software that are central to the research but not yet described in published literature, software must be made available to editors and reviewers. We strongly encourage code deposition in a community repository (e.g. GitHub). See the Nature Portfolio guidelines for submitting code & software for further information.

Data

Policy information about availability of data

All manuscripts must include a data availability statement. This statement should provide the following information, where applicable:

- Accession codes, unique identifiers, or web links for publicly available datasets
- A description of any restrictions on data availability
- For clinical datasets or third party data, please ensure that the statement adheres to our policy

The atomic coordinates of the GSDMB-IpaH7.8 complex, GSDMB pore, GSDMB pore without β-barrel have been deposited in the Protein Data Bank (PDB) under accession numbers 8EFP, 8ET2, and 8ET1, respectively. The associated cryo-EM density maps have been deposited in the Electron Microscopy Data Bank (EMDB) under accession numbers EMD-28087, EMD-28584, and EMD-28583, respectively. All other data, for example, the atomic coordinate of GSDMB prepore which is

not deposited because of the low resolution, are available from the corresponding author upon request. Several structural coordinates in the PDB database were used in this study, which can be located by accession numbers 6CB8, 5B5R, 6N9O, 6N9N, 6VFE, 7V8H, and 3CVR.

Human research participants

Policy information about studies involving human research participants and Sex and Gender in Research.

Reporting on sex and gender	N/A
Population characteristics	N/A
Recruitment	N/A
Ethics oversight	N/A

Note that full information on the approval of the study protocol must also be provided in the manuscript.

Field-specific reporting

Please select the one below that is the best fit for your research. If you are not sure, read the appropriate sections before making your selection.

☒ Life sciences ☐ Behavioural & social sciences ☐ Ecological, evolutionary & environmental sciences

For a reference copy of the document with all sections, see nature.com/documents/nr-reporting-summary-flat.pdf

Life sciences study design

All studies must disclose on these points even when the disclosure is negative.

Sample size	Sample sizes were not pre-determined. Cryo-EM images were collected until structures of satisfactory quality were solved, which suggested sufficient sample size. For biochemical and cellular experiments, no information was derived about a population based on sampling, and therefore sample size determination was not necessary.
Data exclusions	In cryo-EM processing, we discarded "junk" particles that could not be classified into useful 3D reconstructions. This is a widely used and accepted practice in the cryo-EM field. No other data were excluded from analysis.
Replication	All experiments were performed independently at three times with similar results, as described in the figure legends.
Randomization	Proteins, liposomes, and cells were randomly allocated to the wells in each experimental group. Other randomization of experimental groups was not relevant to this study, and independent variables were controlled and did not require randomization.
Blinding	Blinding was not performed as subjective analysis was not needed. Each experiment was analyzed using consistent methods. Random allocation and quantitative measurements using various approaches and reaction kits as described in the methods minimized biased assessments.

Reporting for specific materials, systems and methods

We require information from authors about some types of materials, experimental systems and methods used in many studies. Here, indicate whether each material, system or method listed is relevant to your study. If you are not sure if a list item applies to your research, read the appropriate section before selecting a response.

Materials & experimental systems

n/a	Involved in the study	
☐	☒	Antibodies
☐	☒	Eukaryotic cell lines
☒	☐	Palaeontology and archaeology
☒	☐	Animals and other organisms
☒	☐	Clinical data
☒	☐	Dual use research of concern

Methods

n/a	Involved in the study	
☒	☐	ChIP-seq
☒	☐	Flow cytometry
☒	☐	MRI-based neuroimaging

Antibodies

Antibodies used	Anti-ubiquitin (Thermo Fisher Scientific, PA3-16717, Lot:XG344606, 1:1000)<br>Anti-FLAG (Sigma-Aldrich, F1804, Source#: SLCM4081, 1:1000)<br>Anti-HA (Cell Signaling Technology, 3724S, Lot: 9, 1:1000)<br>Anti-actin (Cell Signaling Technology, 3700S, Lot: 20, 1:1000)<br>Peroxidase AffiniPure Goat Anti-Mouse IgG (H+L) (Jackson Immuno Research Inc., 115-035-166, Lot: 155426, 1:5000)<br>Peroxidase-AffiniPure Goat Anti-Rabbit IgG (H+L) (Jackson Immuno Research Inc., 115-035-144, Lot: 163357, 1:5000)
Validation	All antibodies used in this study are commercially available and have been validated by the manufacturers' and/or previous publications.<br>Anti-ubiquitin (https://www.thermofisher.com/antibody/product/Ubiquitin-Antibody-Polyclonal/PA3-16717. PMID: 30547882)<br>Anti-FLAG (https://www.sigmaaldrich.com/US/en/product/sigma/a8592. PMID: 26727110, 25744187, 20980514, 20356955, 19153083, 18403418, etc)<br>Anti-HA (https://www.cellsignal.com/products/primary-antibodies/ha-tag-c29f4-rabbit-mab/3724. PMID: 27043414, 36307403, 36127332, 35918345, 35908039, 35550517, 34819506, 33972784, etc)<br>Anti-actin (https://www.cellsignal.com/products/primary-antibodies/b-actin-8h10d10-mouse-mab/3700. PMID: 36216837, 35831316, 35672408, 35610475, 35588457, 35602949, 35143048, 35487895, 35332119, etc)<br>Peroxidase AffiniPure Goat Anti-Mouse IgG (H+L) (https://www.jacksonimmuno.com/catalog/products/115-035-166. PMID: 36543799, 35505004, 35658004, 36072551, etc)<br>Peroxidase-AffiniPure Goat Anti-Rabbit IgG (H+L) (https://www.jacksonimmuno.com/catalog/products/111-035-144. PMID: 36109647, 36563856, 36543799, etc)

Eukaryotic cell lines

Policy information about cell lines and Sex and Gender in Research

Cell line source(s)	HEK293T cells were obtained from American Type Culture Collection (ATCC).
Authentication	HEK293T cells were authenticated by ATCC.
Mycoplasma contamination	All cell lines were tested to be mycoplasma-negative by PCR.
Commonly misidentified lines<br>(See ICLAC register)	No commonly misidentified cell lines were used in this study.
